# Significant Impacts of Increasing Aridity on the Arid Soil Microbiome

**DOI:** 10.1128/mSystems.00195-16

**Published:** 2017-05-30

**Authors:** Julia W. Neilson, Katy Califf, Cesar Cardona, Audrey Copeland, Will van Treuren, Karen L. Josephson, Rob Knight, Jack A. Gilbert, Jay Quade, J. Gregory Caporaso, Raina M. Maier

**Affiliations:** a Department of Soil Water and Environmental Science, University of Arizona, Tucson, Arizona, USA; b Pathogen and Microbiome Institute, Northern Arizona University, Flagstaff, Arizona, USA; c Graduate Program in Biophysical Sciences, University of Chicago, Chicago, Illinois, USA; d Department of Microbiology and Immunology, Stanford University, Stanford, California, USA; e Departments of Pediatrics and Computer Science and Engineering and Center for Microbiome Innovation, University of California San Diego, San Diego, California, USA; f Department of Surgery, University of Chicago, Chicago, Illinois, USA; g Department of Geosciences, University of Arizona, Tucson, Arizona, USA; Institute of Soil Science, Chinese Academy of Sciences

**Keywords:** arid soil microbiome, climate change, desert, desertification, microbial diversity

## Abstract

Global deserts occupy one-third of the Earth’s surface and contribute significantly to organic carbon storage, a process at risk in dryland ecosystems that are highly vulnerable to climate-driven ecosystem degradation. The forces controlling desert ecosystem degradation rates are poorly understood, particularly with respect to the relevance of the arid-soil microbiome. Here we document correlations between increasing aridity and soil bacterial and archaeal microbiome composition along arid to hyperarid transects traversing the Atacama Desert, Chile. A meta-analysis reveals that Atacama soil microbiomes exhibit a gradient in composition, are distinct from a broad cross-section of nondesert soils, and yet are similar to three deserts from different continents. Community richness and diversity were significantly positively correlated with soil relative humidity (SoilRH). Phylogenetic composition was strongly correlated with SoilRH, temperature, and electrical conductivity. The strongest and most significant correlations between SoilRH and phylum relative abundance were observed for *Acidobacteria*, *Proteobacteria*, *Planctomycetes*, *Verrucomicrobia*, and *Euryarchaeota* (Spearman’s rank correlation [*r*_s_] = >0.81; false-discovery rate [*q*] = ≤0.005), characterized by 10- to 300-fold decreases in the relative abundance of each taxon. In addition, network analysis revealed a deterioration in the density of significant associations between taxa along the arid to hyperarid gradient, a pattern that may compromise the resilience of hyperarid communities because they lack properties associated with communities that are more integrated. In summary, results suggest that arid-soil microbiome stability is sensitive to aridity as demonstrated by decreased community connectivity associated with the transition from the arid class to the hyperarid class and the significant correlations observed between soilRH and both diversity and the relative abundances of key microbial phyla typically dominant in global soils.

**IMPORTANCE** We identify key environmental and geochemical factors that shape the arid soil microbiome along aridity and vegetation gradients spanning over 300 km of the Atacama Desert, Chile. Decreasing average soil relative humidity and increasing temperature explain significant reductions in the diversity and connectivity of these desert soil microbial communities and lead to significant reductions in the abundance of key taxa typically associated with fertile soils. This finding is important because it suggests that predicted climate change-driven increases in aridity may compromise the capacity of the arid-soil microbiome to sustain necessary nutrient cycling and carbon sequestration functions as well as vegetative cover in desert ecosystems, which comprise one-third of the terrestrial biomes on Earth.

## Abstract (precis)

We identify key environmental and geochemical factors that shape the arid soil microbiome along aridity and vegetation gradients spanning over 300 km of the Atacama Desert, Chile. Decreasing average soil relative humidity and increasing temperature explain significant reductions in the diversity and connectivity of these desert soil microbial communities and lead to significant reductions in the abundance of key taxa typically associated with fertile soils. This finding is important because it suggests that predicted climate change-driven increases in aridity may compromise the capacity of the arid-soil microbiome to sustain necessary nutrient cycling and carbon sequestration functions as well as vegetative cover in desert ecosystems, which comprise one-third of the terrestrial biomes on Earth.

## INTRODUCTION

Climate change-driven increases in temperature and aridity threaten the productivity of arid ecosystems (1–10). Desert regions store 27% of soil organic carbon (SOC) reserves (3, 11, 12), and their continued degradation and loss of productivity (13) in response to megadroughts, global warming, and anthropogenic activities have contributed to a current assessment that 24% of land, globally, is degrading (14). Loss of arid ecosystem productivity and function due to severe land degradation, referred to as desertification (2, 8–10), is projected to negatively impact the livelihoods of 250 million people in the developing world (2) and to drive directional shifts toward expansion of arid land areas (8, 15). Desertification is generally defined as a significant and long-term reduction in biological productivity (13); however, there is little consensus concerning the environmental forces that drive this phenomenon (9, 10). Research efforts aimed at mitigating desertification have focused primarily on shifts in above-ground ecosystem structure, aridity indices (7, 8, 16), and soil degradation and nutrient status (6, 9, 17). However, one area that has received little attention is the impact of desertification on the arid-soil microbiome and on how aridity impacts on the microbiome subsequently influence desertification processes (1). The arid-soil microbiome is largely uncharacterized (1, 18), and yet microbial ecosystem services (i.e., biogeochemical cycling) are likely particularly critical in arid regions (1) because of limited macrofaunal and plant biodiversity. Warming trends over arid regions are predicted to be twice as great as over humid regions (8), making these regions highly susceptible to ecosystem degradation in the face of climate change-associated increases in aridity (3, 7).

Biogeographic analysis of global soil microbial communities has revealed that desert microbiomes are phylogenetically and functionally distinct from those of other biomes (19) and contain a lower diversity of functions associated with nutrient cycling. In addition, abiotic deterministic processes have been shown to shape desert soil microbial assemblages (1, 20). To properly evaluate the impact of climate change on arid land productivity, we must determine the resistance of these low-diversity microbiomes and improve our understanding of the potential impacts of increasing aridity. In lieu of prospective longitudinal monitoring, soil microbiome analyses across gradients of aridity represent a first step toward addressing this issue. However, the limited studies that have been performed to date have shown inconsistent results. One analysis of a steep precipitation gradient from desert to forest soils (100 to 900 mm of rainfall year^−1^) showed that a more pronounced vegetation effect on microbial community composition was observed with increasing aridity (21) but that bacterial and archaeal diversity have not been constrained overall by precipitation (22). In contrast, a second survey of global dryland soils covering a similar precipitation range (aridity index = 0.05 to 0.55) found that increasing aridity and the associated indirect effects (i.e., decreased SOC levels) caused reductions in the abundance and diversity of bacteria and fungi (23).

**FIG 1:**
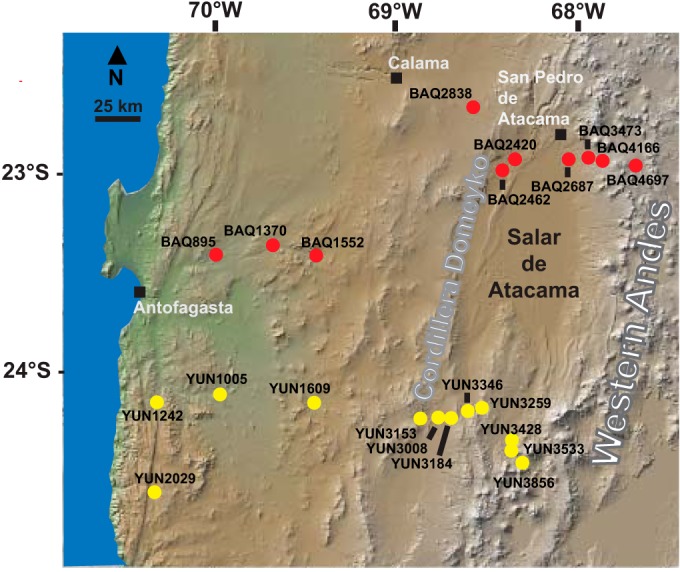
Map of site locations for Baquedano (red) and Yungay (yellow) transects.

In this study, we exploited steep aridity gradients present in the Atacama Desert of northern Chile to identify specific deterministic factors explaining variation in soil microbial communities in the arid and hyperarid subclasses of dryland ecosystems (0 to 120 mm of mean annual precipitation [MAP]). The specific objective was to evaluate environmental and geochemical influences on desert soil microbial community structure and interactions. The environmental parameters evaluated were selected based on previous studies of desert and global soil microbial communities (1, 18, 19, 22–25). Microbial and geochemical profiles from soils along two transects (Fig. 1; see also Table S1 in the supplemental material) were integrated with 3 years of climate data from on-site data loggers. We hypothesize that in arid ecosystems, regions of higher aridity correlate with decreased microbial taxonomic richness and significant changes in phylogenetic composition. Further, we hypothesize that soils with higher average soil relative humidity support denser, more tightly connected communities and that the transition from arid to hyperarid moisture regimes is characterized by a significant decrease in networks of co-occurring taxa within the soil microbiome.

10.1128/mSystems.00195-16.3 TABLE S1  Transect site locations and geochemical and environmental data. Download TABLE S1, PDF file, 0.2 MB. Copyright © 2017 Neilson et al. 2017 Neilson et al. This content is distributed under the terms of the Creative Commons Attribution 4.0 International license.

## RESULTS AND DISCUSSION

Soil samples were collected in March 2012 from two parallel west-east elevational transects traversing the Atacama Desert (250 to 300 km) from the Pacific Ocean near Antofagasta to the western slopes of the Andes near the Argentinian border (Fig. 1; see also Table S1 in the supplemental material). The transects begin on the hyperarid central plateau that extends in an area that is 1,000 to 2,000 m above sea level (masl), has been devoid of vegetation for millions of years, and receives virtually no precipitation (<5 mm of MAP) (26–28). The transects then extend east to arid regions located above 3,000 masl on the western slopes of the Andes that support consistent perennial vegetation with 36 to 115 mm of MAP (29, 30). Twelve sites were located along a southern transect, referred to as Yungay (YUN; Antofagasta to Paso de Socompa), and 10 sites were included in the northern transect, referred to as Baquedano (BAQ; Baquedano to Paso Jama). At each site, percent plant cover, geochemical measurements, and soil relative humidity and temperature were recorded as explained in Materials and Methods. Triplicate soil pits were sampled for characterization of microbial community composition (16S rRNA amplicon sequencing) and soil organic carbon (SOC; Table S2). SOC levels ranged from 0.17 to 16.45 mg C g dry soil^−1^, with the highest values associated with soils of the most vegetated sites (Table S1).

10.1128/mSystems.00195-16.4 TABLE S2  Bacterial and archaeal diversity indices, DNA extract concentrations, and soil organic carbon concentrations for each sample included in the microbial analysis. Download TABLE S2, PDF file, 0.1 MB. Copyright © 2017 Neilson et al. 2017 Neilson et al. This content is distributed under the terms of the Creative Commons Attribution 4.0 International license.

Nitrate and sulfate levels exceed 20 and 500 µmol·g^−1^ dry soil, respectively, at shallow levels of many soils in the Atacama (see Fig. S1 in the supplemental material) and are probably the best indicators of long-term hyperaridity at these sites. Observed salt abundances take thousands of years to accumulate (31) and generally require mean annual rainfall levels of <1.0 cm·year^−1^ (32), fitting the definition of “hyperarid” on the United Nations Environment Programme (UNEP) aridity index (31). Elevation and rainfall in the Atacama Desert are closely linked, and abundant soil salt and, hence, hyperarid conditions are confined to <3,150 masl along the YUN transect and to <2,500 masl along the moister BAQ transect. In this study, sample sites were classified into three aridity classes: hyperarid, margin, and arid. The sites classified as hyperarid (on the basis of nitrate and sulfate profiles) were YUN1005, YUN1242, YUN1609, YUN2029, YUN3153, BAQ895, BAQ1370, and BAQ1552. High sulfate levels at sites YUN3008 and YUN3184 are controlled by hydrologic conditions associated with the Salar de Imilac region rather than long-term aridity; thus, these sites were not classified as hyperarid. In this study, sites with vegetation present at the time of sampling in 2012 were classified as arid (Table S1) and all sites with neither salt accumulation nor vegetation present were classified as margin. Unsurprisingly, levels of vegetation are closely tied to the presence of higher rainfall and salt-depleted soils. During the years of this study, live vegetation was present above ~2,600 masl only at the BAQ transect sites and above ~3,250 masl only for the YUN transect (Table S1).

10.1128/mSystems.00195-16.1 FIG S1  Soil nitrate and sulfate concentrations at 10-cm depth increments from 0 to 50 cm for all sites of Baquedano (top) and Yungay (bottom) transects. Salt abundances at shallow depths in Atacama soils are indicators of long-term hyperaridity. Download FIG S1, JPG file, 1.1 MB. Copyright © 2017 Neilson et al. 2017 Neilson et al. This content is distributed under the terms of the Creative Commons Attribution 4.0 International license.

### Environmental controls of community diversity and phylogenetic composition.

Profiles of bacterial and archaeal communities at each site were generated using the v4 hypervariable region of the 16S rRNA bacterial and archaeal genes following a modification of the Earth Microbiome Project protocols (33–35) as described in Materials and Methods. A total of 40 sample pits from 16 sites (of the original 22) generated high-quality data for downstream analysis (Table S2). Sequence information and missing data points from the original 22 sites are explained in Materials and Methods.

**FIG 2:**
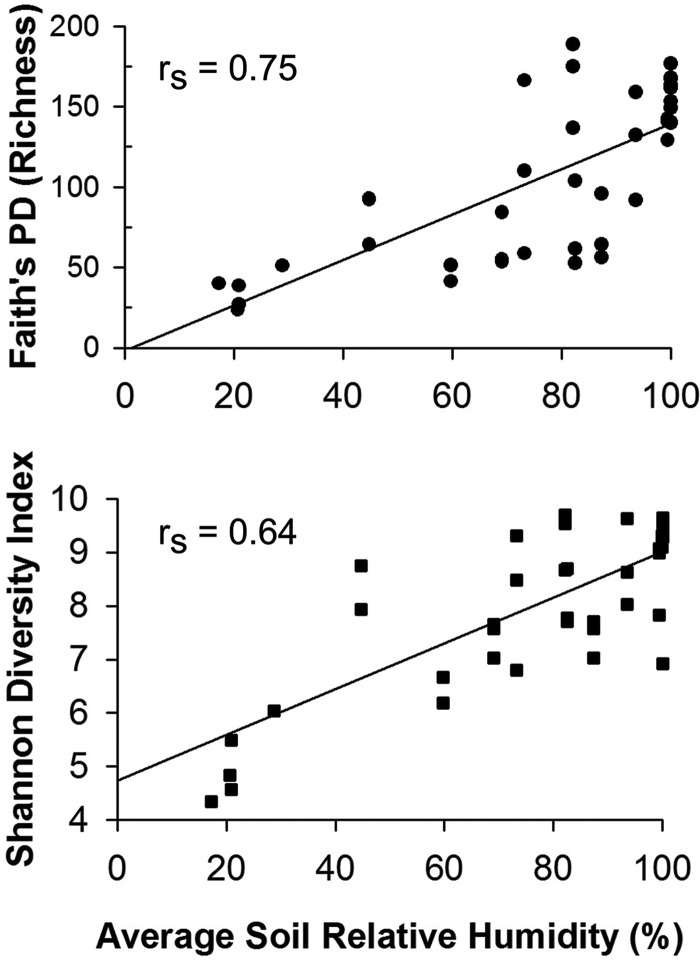
Correlation between average soil relative humidity and microbial richness (PD) (top panel) and diversity (Shannon index) (bottom panel); *r*_s_ = Spearman’s rank correlation, *P* < 0.0001.

Microbial community richness (Faith’s phylogenetic diversity [PD]; *r*_s_ = 0.75; *P* < 0.0001) and diversity (Shannon index; Spearman’s rank correlation [*r*_s_] = 0.64; *P* < 0.0001) decreased significantly with decreasing average soil relative humidity (AvgSoilRH; Fig. 2; Table S1). A 7-fold reduction in community PD (richness) and 51% decrease in Shannon diversity (Table S2) were observed from the arid site with the greatest diversity (BAQ4166; PD = 169 ± 6.9) to the hyperarid site with lowest diversity (YUN1005; PD = 23.6). In addition, richness was significantly greater in vegetated sites than in unvegetated sites (*t* test, *P* < 0.0001). Maestre et al. (23) also found that increases in aridity were linearly associated with reductions in bacterial diversity (Shannon index) for dryland soils; however, the correlation was weak (*r*^2^ = 0.157), suggesting that impacts of increasing aridity on the soil microbiome are amplified in ecosystems that are more arid.

**FIG 3:**
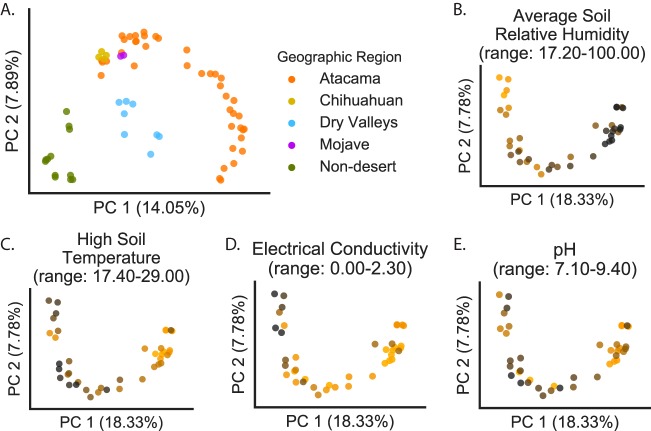
Unweighted UniFrac PCoA plots. (A) Combined data from the present Atacama Desert study and from Fierer et al. (19). (B to E) Atacama-only data. (A) Samples colored by their geographic origins. (B to F) Samples colored by soil properties, where light colors indicate the low end of the specified range and dark colors indicate the high end of the specified range. These data illustrate statistical results presented in Table S3, specifically, that average soil relative humidity is a better predictor of sample composition in the Atacama Desert than high soil temperature, electrical conductivity, or pH.

A beta diversity meta-analysis using unweighted UniFrac-based principal-coordinate analysis (PCoA; Fig. 3A) was used to compare the composition of the Atacama Desert microbial communities to those of a cross-biome survey of global soil samples from Fierer et al. (19). The plots reveal that soil communities from the Atacama represent a gradient in microbial beta diversity that is clearly distinct from the results seen with globally distributed nondesert soils that included tropical forests in Peru and Argentina and boreal and temperate forests, grasslands, and tundra in North America. In contrast, the Atacama soil communities are similar in composition to those of three diverse global deserts and fit into the classification of Fierer et al. systematically separating desert from nondesert soils (19). The Atacama communities with higher relative humidity overlap along PC1 with those from the Chihuahuan and Mohave deserts of the United States, and the communities from the drier Atacama sites overlap along PC2 with those from the McMurdo Dry Valleys of Antarctica. A nonmetric multidimensional scaling (NMDS) analysis of the same data showed a similar relationship between desert and nondesert soil communities (Fig. S2). Previous limited surveys of the Atacama Desert identified significant differences in the soil bacterial communities of hyperarid and arid regions (24); however, the continuous gradient in phylogenetic composition observed in this study has not been previously documented. A comparison of UniFrac distance matrices with environmental variables identified AvgSoilRH as a strong driver of both qualitative and quantitative phylogenetic community composition (Fig. 3B) (unweighted UniFrac Mantel’s *r* = 0.625; false-discovery rate [FDR {*q*}] = 0.0001; weighted UniFrac Mantel’s *r* = 0.574; *q* = 0.0001; Table S3). Weighted and unweighted profiles were highly correlated for both the Atacama and Fierer samples (see Materials and Methods); thus, unweighted correlations are primarily discussed. Significant but weaker correlations were also observed for high soil temperature (HighSoilTemp; Fig. 3C) (Mantel’s *r* = 0.337; *q* = 0.0001; Table S3) and soil electrical conductivity (EC; Fig. 3D) (Mantel’s *r* = 0.496; *q* = 0.0001; Table S3). Interestingly, the correlation with pH as an explanatory variable was weak (Fig. 3E) (UniFrac Mantel’s *r* = 0.176 and *q* = 0.010 [unweighted] and *r* = 0.108 and *q* = 0.063 [weighted]; Table S3). pH has been shown to explain variations in phylogenetic microbial community composition for diverse terrestrial soils (36); however, for these neutral to alkaline desert soils (Table S1), pH was not a significant factor. This potentially indicates resistance to alkaline pH in populations of the arid soil microbiome. Finally, the presence of vegetation had a significant association with microbial community composition (analysis of similarity [ANOSIM] *R* = 0.617; *P* = 0.001), although the results corresponding to the percentages of plant cover were not significant (Mantel’s *r* = 0.184; *q* = 0.018).

10.1128/mSystems.00195-16.2 FIG S2  NMDS plots for data analyzed by PCoA in Fig. 3. (A) Combined data from the present Atacama Desert study and from Fierer et al. (19). (B to E) Atacama-only data. (A) Samples are colored by their geographic origin, indicating that nondesert soils cluster apart from desert soils from diverse locations; stress factor = 0.054. (B to F) Samples are colored by soil properties, where light colors indicate the low end of the specified range and dark colors indicate the high end of the specified range; stress factor = 0.051. These data illustrate the statistical results presented in Table S3, specifically, that average soil relative humidity is a better predictor of sample composition in the Atacama Desert than high soil temperature, electrical conductivity, or pH. Download FIG S2, EPS file, 1.1 MB. Copyright © 2017 Neilson et al. 2017 Neilson et al. This content is distributed under the terms of the Creative Commons Attribution 4.0 International license.

10.1128/mSystems.00195-16.5 TABLE S3  UniFrac Mantel’s correlation values between UniFrac distance matrix and the pairwise dissimilarity for each environmental variable. Download TABLE S3, PDF file, 0.2 MB. Copyright © 2017 Neilson et al. 2017 Neilson et al. This content is distributed under the terms of the Creative Commons Attribution 4.0 International license.

A BEST analysis (37) was performed to identify the variable (or combination of explanatory variables) from among the parameters evaluated in our study that best explained the variance in phylogenetic composition of the Atacama microbial communities (Table 1). The combination of AvgSoilRH, HighSoilRH, HighSoilTemp, elevation, and EC best explained the variability (Spearman’s *r*_s_ = 0.776) in community composition. For this study, elevation was synonymous with site location; thus, communities sampled from the same site were more similar to each other than to those from other sites. As explained previously, EC or soil salinity is strongly influenced by precipitation and all sites with high EC values were associated with the hyperarid class or Salars (Table S1). Due to the significance of EC, a secondary analysis was done to evaluate the significance of nitrate and sulfate in the hyperarid sites (Fig. S1). Using values from the depth of 10 to 20 cm from which microbial samples were collected, both unweighted and weighted UniFrac distances correlated moderately with nitrate (Mantel’s *r* = 0.459 and *q* = 0.003 [unweighted] and Mantel’s *r* = 0.503 and *q* = 0.0045 [weighted]) and weakly with sulfate (Mantel’s *r* = 0.307 and *q* = 0.014 [unweighted] and Mantel’s *r* = 0.311 and *q* = 0.01 [weighted]); however, these correlations were more than an order of magnitude less significant than those seen with EC (Table S3). Nitrates and sulfates were consistently present at depth at all hyperarid sites, but their concentrations at the depth of 10 to 20 cm differed significantly between hyperarid sites (Fig. S1). At shallow depths, levels of nitrates were high only for YUN1242 and YUN1005 and levels of sulfates were high only for YUN1005 and YUN3153. Nitrate and sulfate accumulation at YUN2029 and YUN1609 was found only at or below 30 cm and 20 cm, respectively. In contrast, AvgSoilRH was low across all hyperarid sites (Table S1) and thus represented an environmental variable that explained the unique composition of the hyperarid microbial communities that was more consistent and significant than the presence of nitrates or gypsum. We are currently conducting studies to evaluate potential influences of nitrate and sulfate concentration on the phylogenetic composition and activity of microbial communities at each of the hyperarid sites.

**TABLE 1:** BEST analysis identifying combinations of sample variables best explaining variance in soil microbiome phylogenetic composition^a^

Environmental variable(s) (no. of variables)	Spearman’s coefficient (*r*_s_)
E, EC, AvgSoilRH, HighSoilRH, HighSoilT (5)	0.776
E, EC, AvgSoilRH, HighSoilRH, PercSoilRH100, HighSoilT (6)	0.775
E, EC, HighSoilRH, HighSoilT (4)	0.772
E, EC, AvgSoilRH, HighSoilRH, PercSoilRH100, HighSoilTemp, LowSoilT (7)	0.770
EC, HighSoilRH, AvgSoilT (3)	0.764
E, EC, AvgSoilRH, HighSoilRH, PercSoilRH100, AvgSoilT, HighSoilT, LowSoilT (8)	0.760
E, pH, EC, AvgSoilRH, HighSoilRH, PercSoilRH100, AvgSoilTemp, HighSoilTemp, LowSoilTemp (9)	0.744
E, EC (2)	0.728
E, pH, EC, AvgSoilRH, HighSoilRH, LowSoilRH, PercSoilRH100, AvgSoilT, HighSoilT, LowSoilT (10)	0.726
E, pH, SOC, EC, AvgSoilRH, HighSoilRH, LowSoilRH, PercSoilRH100, AvgSoilT, HighSoilT, LowSoilT (11)	0.712
E, pH, SOC, EC, AvgSoilRH, HighSoilRH, LowSoilRH, PercSoilRH100, AvgSoilT, HighSoilT, LowSoilT, % vegetation cover (12)	0.687
HighSoilRH (1)	0.663

a E, elevation; EC, electrical conductivity; AvgSoilRH, average soil relative humidity; HighSoilRH, high soil relative humidity; HighSoilT, high soil temperature; PercSoilRH100, percentage of RH values at each site that represented 100% RH; LowSoilT, low soil temperature; AvgSoilT, average soil temperature; LowSoilRH, low soil relative humidity; SOC, soil organic carbon. All high and low values are averaged over 7 days.

The significant correlation of AvgSoilRH and HighSoilTemp with phylogenetic composition within the Atacama Desert soil microbiome is highly relevant because both factors will continue to be influenced by climate change in arid regions (3, 5, 7). In summary, the transects presented here provide a gradient of increasing aridity and the results strongly suggest that climate change-driven increases in aridity may significantly impact the phylogenetic composition, community richness, and diversity of desert soils.

### Increased aridity correlates with network topology shifts.

**FIG 4:**
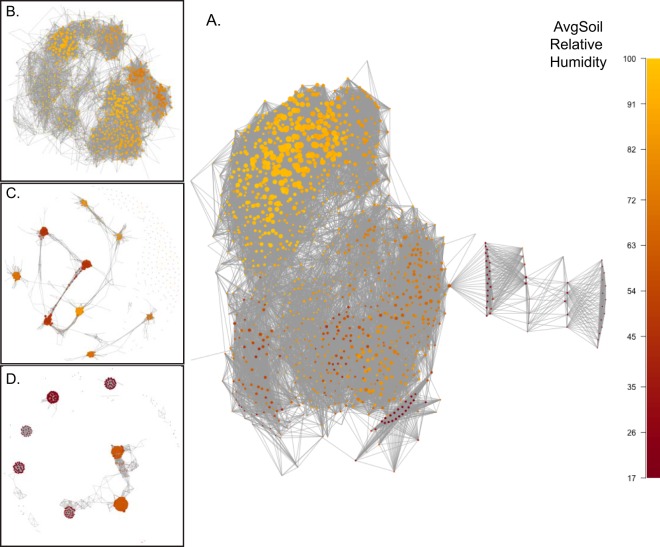
Network topology plots of Atacama microbial communities. (A) Full Atacama desert network. (B) Arid subnetwork. (C) Margin subnetwork. (D) Hyperarid subnetwork. Node colors represent average levels of soil relative humidity of the site or sites where the OTU is located, and the relative node sizes indicate the degrees of the node OTU. Subnetworks were created individually.

Co-occurrence patterns can be used to identify important interactions among members of a microbiome (38, 39). We employed network-based analysis to evaluate the impact of soilRH on the network structure of correlations in the relative abundances (RAs) of taxa. The data from the general Atacama desert network (Fig. 4A) reveal that operational taxonomic units (OTUs) (network nodes) primarily associated with higher relative humidity sites (yellow) are involved in networks that are more densely connected than those associated with lower relative humidity sites (brown). Greater than 99% of the co-occurrences in the Atacama desert network were positive (correlation thresholds, ≥0.81), indicating that correlated microorganisms had similar responses to environmental conditions. The degree of each OTU (size of node) represents the number of taxa in the community that co-occur with that OTU, and it is evident that OTUs primarily associated with sites with higher relative humidity (yellow) have a higher degree (larger circle) on average than OTUs in sites with lower relative humidity (brown). Spearman’s rank correlation analysis revealed significant (*P* < 0.001) and strong positive correlations between AvgSoilRH and node degree (*r*_s_ = 0.76), node betweenness (*r*_s_ = 0.66), edgecount (*r*_s_ = 0.78), and size (*r*_s_ = 0.72) (betweenness values represent the centrality of a node [OTU] with respect to other members of the community [i.e., quantify the number of times that node functions as a bridge on the shortest path connecting two associated nodes], edgecount values represent the number of co-occurrences, and size values quantify the number of OTUs; thus, communities in regions of higher soilRH contained more OTUs with correlated relative abundance patterns than communities in progressively drier areas). It must be noted that network analysis of soil microbial communities is limited by the fact that soils represent heterogeneous substrates for microbial colonization; thus, we cannot confirm that co-occurring phylotypes are actually in physical contact with one another in the environment. However, on the basis of the observed gradients in network metrics, soils with higher AvgSoilRH appear to support denser, more tightly connected communities.

To test this observation, subnetworks were generated for each of the three aridity classes: arid (Fig. 4B), margin (Fig. 4C), and hyperarid (Fig. 4D). The subnetwork topologies reveal a striking breakdown in community cohesion in the transition from the arid class to the hyperarid class. The arid subnetwork retained an integrated topology similar to that of the full network; however, OTUs from the margin sites were located in loosely connected clusters and the hyperarid topology revealed a significant loss of graph cohesion that we term the “island effect.” Since vertex betweenness and edge count are metrics sensitive to the network size (number of OTUs), they are not suitable for comparisons of the different sized subnetworks. Therefore, global network metrics that are normalized by their size such as average path length and betweenness centralization were used to quantify this apparent loss of network cohesion in the subnetworks. Average path length data represent graph-level quantification of the shortest path lengths between all nodes in a network. Thus, dense, tightly assembled graphs have lower average path length scores than less-cohesive graphs due to the close proximity of nodes within the network. Betweenness centralization is a graph-level measure derived from the betweenness centrality scores of the individual nodes within the network. The island topology is characterized by a bridge and hub effect where the bridges are paths connecting islands and the hubs are connector nodes that connect the bridges between island clusters. The role of hubs as points of connection gives them high centrality or betweenness scores that are frequently manyfold higher than those of other nonhub network nodes. As with the average path length, betweenness centralization scores are higher for the island topoplogy than for more-cohesive graphs. Evaluation of sites from each aridity class (Table 2) reveals that the median average path length is shortest for arid communities and longest for the hyperarid communities. The same pattern was observed for betweenness centralization, where the median value was highest for the hyperarid communities. Thus, arid soils support denser, more tightly connected communities than hyperarid soils. This could be interpreted as suggesting that microbes have greater codependency, with respect to either dependency on each other or dependency on shared resources, in arid soils than in hyperarid soils. Whether this codependency is representative of metabolic interactions between taxa remains unknown.

**TABLE 2:** Network statistics associated with distinct Atacama Desert aridity classes and modified arid and margin communities randomly sampled to a community richness level equal to the hyperarid community^a^

Network ID	Size	Degree	Betweenness	Edge count	Avg path length	Betweenness centralization
Original Atacama communities						
Arid	559	23.98	634.38	7408	3.51	0.04
Margin	357	15.63	476.23	2852	3.69	0.06
Hyperarid	122	11.34	124.92	562	4.36	0.11
Community richness modified to the hyperarid level						
Arid	131	10.4	83.85	698	2.90	0.07
Margin	119	10.8	72.9	680	2.90	0.08

a Median values are reported for each aridity class. Modified values represent averages of 500 simulations.

Community connectivity may be impacted by network size (i.e., community richness), and our arid, margin, and hyperarid soils differed in their size data. We therefore wanted to test whether the decrease that we observed in community connectivity is driven by a decrease in diversity rather than in the aridity of the environment. To this end, we performed a simulation where we artificially modified the arid and margin community richness to equal the median richness of the hyperarid communities. By removing the diversity richness effect, we can test if the decrease that we observed in community connectivity would remain in the simplified networks as a function of the aridity change in the environment. Also, since some connectivity metrics (betweenness and edge count) are impacted by network size, the simulated standardization of network size allowed a fair comparison of all connectivity metrics. The modified arid and margin communities were created through random sampling of the original communities. Networks were then created from each modified community (500 repetitions), the network statistics were averaged at the sample level, and the median value per climate type was computed (Table 2). The modified arid and margin communities then had size and degree values comparable to those corresponding to the original hyperarid network (Table 2), but the average path length, betweenness centralization, and betweenness values of the modified arid and margin communities were lower than those of the original, unmodified hyperarid community (Table 2). In addition, the median edge count was higher for both arid and margin aridity classes, indicating that even after adjustment of community size, these communities retained more correlated co-occurrences between OTUs than the hyperarid communities. This illustrates that while the degree data remained unchanged within networks of similar sizes, betweenness, edgecount, average path length, and betweenness centrality data did not. The arid and margin communities therefore retain higher connectivity than the hyperarid community, even when their richness is artificially modified to the level of the hyperarid community.

In conclusion, the data suggest that the decrease in community connectivity is not simply an artifact of reduced community richness. Rather, increasing aridity correlates with a decrease in connectivity in the microbial communities of desert soils. The impact of the topology transition (from cohesive network to island confirmation) on community functional potential is unknown. However, greater connectivity within microbial assemblages may be particularly important for nutrient-poor communities, in which interconnected groups of taxa have been shown to exchange metabolites to enhance survival (40).

Analysis of the topology of the hyperarid subnetwork (Fig. 4D) provides an intriguing tool for probing specific assemblages of novel microbial communities. The hyperarid soils are characterized by community profiles dominated by *Actinobacteria* (67% to 86% relative abundance [RA]), *Chloroflexi* (4.2% to 9.7% RA), *Proteobacteria* (2.2% to 15.9% RA), and *Gemmatimonadetes* (0.5% to 7.9% RA). This distribution is similar to that observed previously in hyperarid regions of the Atacama (24, 25, 41, 42) as well as in global deserts, including the McMurdo Dry Valleys of Antarctica and the Tataouine Desert (18). An intriguing pattern emerged from the analysis of Atacama hyperarid sites. Communities were heavily dominated by just one or two taxa (22% to 45% RA) with distributions that varied significantly by geographic location. Combining these data with the OTU profiles of individual network clusters, we see, for example, a *Chloroflexi* strain of class TK10 and order AKYG885 with high (1.2% to 3.5%) abundance in four of the five hyperarid locations but with lower (<1%) relative abundance in all but one nonhyperarid site. Within the hyperarid network, this taxon was associated with just four of the seven hyperarid clusters. Hyperarid regions of the Atacama harbor an abundance of rare taxa with unknown functional potential (18). The combined use of the network co-occurrence patterns and location-specific environmental profiles associated with a specific rare taxon such as this *Chloroflexi* strain can be used to guide future enrichment efforts focused on identifying the functional potential of abundant and novel desert microbes.

### Correlations between abundances of key soil taxa and increasing aridity.

**FIG 5:**
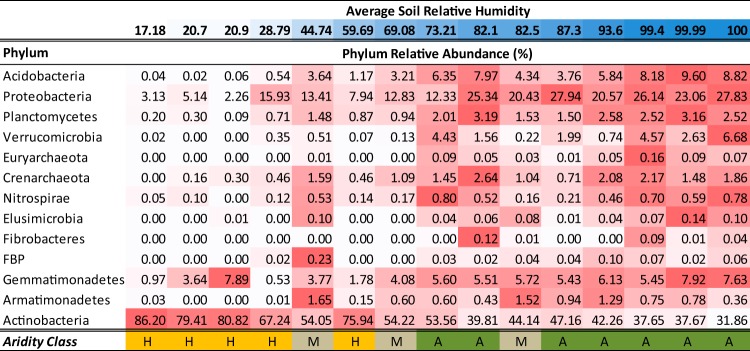
Relative abundance (RA) of individual phyla that are strongly correlated (|*r*_s_| = >0.6; *q* = <0.02) with average soil relative humidity (AvgSoilRH). Phyla are listed by decreasing strength of positive correlation (*r*_s_ = 0.93 to −0.95; Table S4) with RA listed according to the AvgSoilRH of the transect site. *Actinobacteria* data are negatively correlated (*r*_s_ = −0.95; *q* = 2.3 × 10^−15^) with AvgSoilRH. The heat map is normalized within each phylum to the maximum RA (dark red) for that phylum. Analysis is limited to phyla with site RA = >0.1%. Aridity class labels: H, hyperarid; M, margin; A, arid (as defined in the text).

Phylogenetic profiles of all samples were analyzed to identify the bacterial and archaeal taxa most impacted by AvgSoilRH (Fig. 5; Table S4). Surprisingly, *Archaea* relative abundance correlated strongly with AvgSoilRH (*r*_s_ = 0.757) and the domain was undetectable at the most arid site (Fig. 5). Strong correlations were observed for both *Euryarcheota* (*r*_s_ = 0.818, *q* = 0.0007) and *Crenarchaeota* (*r*_s_ = 0.757, *q* = 0.005). All *Euryarcheota* belonged to the *Thermoplasmata* class of order E2, and all *Crenarchaeota* belonged to the genus “*Candidatus* Nitrososphaera.”

10.1128/mSystems.00195-16.6 TABLE S4  Spearman’s rank correlations between average soil relative humidity and relative abundances of taxa at the domain and phylum levels. Download TABLE S4, PDF file, 0.2 MB. Copyright © 2017 Neilson et al. 2017 Neilson et al. This content is distributed under the terms of the Creative Commons Attribution 4.0 International license.

Thirteen bacterial phyla and candidate phyla were also significantly correlated with decreasing AvgSoilRH levels (*q* = <0.05; Table S4). The strongest correlations were observed for *Acidobacteria* (*r*_s_ = 0.925), *Proteobacteria* (*r*_s_ = 0.857), *Plantomycetes* (*r*_s_ = 0.857), *Verrucomicrobia* (*r*_s_ = 0.842), *Nitrospirae* (*r*_s_ = 0.764), and *Elusimicrobia* (*r*_s_ = 0.702). Decreases in RA were linear for *Acidobacteria* (*r*^2^ = 0.80), *Proteobacteria* (*r*^2^ = 0.75), and *Planctomycetes* (*r*^2^ = 0.76), with the arid communities characterized by 5- to 133-fold greater RAs of these taxa than the hyperarid locations (Fig. 5). In contrast, *Actinobacteria* (*r*_s_ = −0.95) abundance had a strong negative correlation with AvgSoilRH (Fig. 5; Table S4). Phyla not correlated with AvgSoilRH included *Firmicutes*, *Bacteroidetes*, *Cyanobacteria*, GAL15, and *Chloroflexi*. As a point of reference, multiple cross-biome surveys have identified *Acidobacteria*, *Proteobacteria*, *Actinobacteria*, *Verrucomicrobia*, and *Bacteroidetes* to be the dominant phyla in soil communities (19, 36, 43). Thus, increasing aridity in the Atacama region correlates with significant decreases in the RA of three of the five dominant phyla of soil microbiomes.

Nitrogen is a limiting nutrient in desert ecosystems, and microbially mediated nitrogen cycling has been shown to be negatively impacted by decreases in microbial community diversity in soils (44). The impact of decreasing AvgSoilRH on 12 genera with taxa known to be associated with either N_2_ fixation or nitrification (Table S5) was investigated to determine the potential impact of increasing aridity on microbially mediated ecosystem services. Decreasing AvgSoilRH levels had a significant impact on the RA of two putative N_2_ fixers, *Bradyrhizobium* (0% to 1.2% RA; *r*_s_ = 0.787, *q* = 0.002) and *Mesorhizobium* (0% to 0.15% RA; *r*_s_ = 0.707, *q* = 0.007); one ammonia oxidizer, *Nitrososphaera* (0% to 2.6% RA; *r*_s_ = 0.757, *q* = 0.006); and one nitrite oxidizer, *Nitrospira* (0% to 0.1% RA; *r*_s_ = 0.805, *q* = 0.002). *Nitrobacter*, *Nitrosomonas*, and *Nitrosospira*, common contributors to nitrification, were not detected at any site. Putative broadly distributed N_2_ fixers *Frankia*, *Sinorhizobium*, *Rhizobium*, and *Azospirillum* (45, 46) were detected in just 2 to 5 of the 16 sites at ≤0.1% RA. These results suggest that the RAs of known N-cycling taxa are significantly diminished by increasing aridity in desert soils. Future research must address whether the RA of known N-cycling taxa correlates with the soil N-cycling capacity of these soils or whether desert soils harbor a novel diversity of N-cycling taxa, such as the *Pontibacter* spp. belonging to *Bacteroidetes* that were isolated from the Taklamakan Desert (China) and harbored both the *nifH* gene and nitrogenase activity (47).

10.1128/mSystems.00195-16.7 TABLE S5  Distribution of soil genera commonly associated with nitrogen-cycling activities and their correlation with average soil relative humidity. Download TABLE S5, PDF file, 0.2 MB. Copyright © 2017 Neilson et al. 2017 Neilson et al. This content is distributed under the terms of the Creative Commons Attribution 4.0 International license.

### Conclusions.

The Atacama Desert soil microbiome is distinct from microbiomes of nondesert soils but similar to microbiomes of other global deserts. A broad gradient in microbial diversity and phylogenetic composition was observed that correlated strongly with soil RH and temperature rather than with pH, a factor previously identified as a significant explanatory variable for diversity in global soils (36). Specifically, increasing aridity correlated with significant decreases in diversity and the RA of key phyla that are typically dominant in fertile soils, as well as in functional guilds associated with N cycling. In addition, network analysis revealed that arid microbial communities were characterized by more densely connected networks than those of the hyperarid communities, whose networks resembled an island topology. The significance of the observed association between increasing aridity and decreasing network connectivity is unknown, but it could have implications for the resilience or ecological function of the respective microbial communities (38, 40). Co-occurrence patterns in soils have been associated with groups of microbes sharing similar ecological niches (38); thus, the hyperarid island topology may indicate a distinct ecological structure for hyperarid soils in which microbial communities separate into isolated assemblages. Further characterization of these assemblages may prove to be a valuable resource for applications ranging from managing desertified regions to guiding the search for extraterrestrial life on planets such as Mars, where recent evidence of hydrated salts (similar to some of those in the Atacama region) suggests the ephemeral presence of surface water (48). In summary, the data suggest that long-term increases in aridity may compromise the stability and genetic potential of the arid soil microbiome. Future research will address whether the novel assemblages that characterize increasingly arid soils harbor an undiscovered biogeochemical-cycling potential important to ecosystem function. The answer has critical implications for the development of new technologies designed to restore productivity to desert ecosystems degraded by megadrought and global warming.

## MATERIALS AND METHODS

### Transect description and sample collection.

Sample sites for the current study were located along two 250-km to 300-km west-east transects traversing the dry hyperarid central region of the Atacama Desert, Chile, and terminating on the arid vegetated western slopes of the Andes (Fig. 1). The southern transect, referred to as Yungay (YUN), extended from site YUN1242 near Varillas along Highway 5 south of Antofagasta (24.141S, 70.312W), passing the Salar de Imilac and ending at YUN3856 (24.446S, 68.296W) in the Andes at the Paso de Socompa border with Argentina. The northern transect, referred to as Baquedano (BAQ), began south of Baquedano along Highway 5 at BAQ895 (23.403S, 69.987W) and continued east, passing south of Calama and San Pedro de Atacama to the BAQ4697 site (22.951S, 67.689W) in the Andes along Highway 27. Both transects crossed the Domeyko mountains (Cordillera Domeyko). Global Positioning System (GPS) coordinates and elevations for all sites are listed in Table S1 in the supplemental material. All sites were sampled from 2 March to 20 March 2012. At each site, plant cover was determined using a rapid survey method for desert plant communities adapted from the Braun-Blanquet method by McAuliffe (49). Geochemical, temperature, and relative humidity parameters for each site were determined from a 50-cm-deep soil pit. Soil samples were collected at 10-cm depth increments to a depth of 50 cm. All soil samples were analyzed for pH, electrical conductivity (EC), nitrate, and sulfate. Following sampling, Hobo U23 Pro v2 temperature and relative humidity data loggers were installed at a depth of 20 cm. The data loggers recorded at 2-h increments from March 2012 to January 2015 (Onset Data Loggers, Bourne, MA) except where noted (Table S1). Two additional pits were dug at each site located 10 m from the original pit. Soil samples from the original pit were collected for microbial analysis and from the two additional pits to provide triplicate samples for microbial analysis. Microbial samples were collected using sterile instruments from the pit sidewall at a depth of 10 to 20 cm. Soil organic carbon (SOC) analysis was performed on all of the samples collected for microbial analysis. Samples to be analyzed for microbial community and SOC were stored on ice and transported as described previously (18). All statistical analyses evaluating the effect of environmental parameters on the soil microbiome used data from samples of soil collected at a depth of 10 to 20 cm. Site BAQ4697 was bulldozed in our absence and the data logger lost; thus, no environmental data were recovered for this site.

### Soil analysis.

Soils were dried and sieved (2 mm pore size) prior to analysis. Soil pH was determined from a 1:1 soil-to-distilled water (dH_2_O) slurry after 1 h of shaking followed by 1 h of rest. EC was determined from the supernatant of a 2:1 dH_2_O soil suspension following 30 min of shaking. Dry soils were subjected to ball milling prior to analysis for sulfate, nitrate, and SOC levels. SOC levels were determined manometrically by high-temperature combustion after pretreatment with 3 N HCl (detection limit, 20 μg). Soil sulfate and nitrate were extracted in a 1:20 soil-to-dH_2_O solution with 24 h of shaking at room temperature. Concentrations of sulfate and nitrate in soil extracts were quantified by ion chromatography using a Thermo Scientific Dionex model ICS-1000 system (Dionex Corp., Sunnyvale, CA), column set AG+AS-22, and a sodium carbonate/bicarbonate eluent.

### Microbial community analysis.

Total genomic DNA was extracted from 0.5-g soil samples using a FastDNA spin kit for soils (MP Biomedicals, LLC, Solon, OH) with modifications to enhance DNA recovery from low-biomass samples (50). Template DNA was quantified with a TBS-380 Fluorometer (Turner Biosystems, Sunnyvale, CA) and Pico green dye (Invitrogen, Carlsbad, CA). Samples with DNA concentrations below the level of detection were reextracted using 2× to 4× replicate extractions (1.0 to 2.0 g soil) that were combined on a FastDNA spin kit binding matrix. Samples still generating no detectable DNA were eliminated from the study, resulting in the removal of 7 samples as follows: one sample pit from each of sites BAQ895, BAQ1370, YUN1005, YUN1609, and YUN3184 and two sample pits from site BAQ1552. DNA extract concentrations are reported for all other samples (Table S2).

The v4 region of the 16S rRNA gene was amplified from all community DNA extracts using bar-coded primers 515F/806R, targeting bacteria and archaea, following a modification of Earth Microbiome Project protocols (33–35). Amplicon sequencing was performed at Argonne National Laboratories using an Illumina MiSeq system and MiSeq control software version 2.2.0. Sequence reads were analyzed using QIIME version 1.9.1 UCLUST-based (51) open reference OTU picking workflow with default parameters unless noted (52). The average number of sequences per sample was 56,323.5 ± 41,366.6, with the minimum number of sequence reads required to retain a sample in the study set at 16,660 (to maximize the number of samples retained). Samples were excluded due to insufficient sequence reads from the following sites: BAQ895, BAQ1370. BAQ1552, YUN1005, YUN1242, YUN1609, YUN2029, YUN3008, YUN3142, and YUN3153. Reads from the 40 remaining samples (Table S2) were clustered into operational taxonomic units (OTUs) at 97% similarity using UCLUST-based open-reference picking against the Greengenes 13_8 reference database preclustered to 97% identity (version 13_5) (51, 53, 54). Representative sequences were then aligned with PyNAST (52), and a phylogenetic tree was constructed with FastTree (55) for phylogenetic diversity calculations. In summary, microbial data from 40 of the original 66 samples, representing 16 of the original 22 transect sites, were analyzed (Table S2). Sites eliminated due to insufficient DNA or sequence reads included BAQ895, BAQ1370, BAQ1552, YUN3008, and YUN3184. Site BAQ4697 was also eliminated due to the loss of environmental data from the Hobo data logger.

In comparing our Atacama samples with the samples from Fierer et al. (19), we applied the iterative open-reference OTU picking protocol implemented in QIIME 1.9.1 to pick OTUs for the samples from Fierer et al. This protocol uses the representative sequences from one set of open-reference OTUs (in this case, the Atacama open-reference OTUs described above) along with the reference database (in this case, the Greengenes 13_8 97% OTUs) as the reference database for a subsequent open-reference OTU picking run. The resulting Fierer OTU table and OTU representative sequences were combined with the Atacama OTU table and OTU representative sequences for diversity analyses. This combined-data set was used only for the ordination meta-analyses presented in Fig. 3 and in Fig. S2 in the supplemental material. Our study and that by Fierer et al. differed in the DNA extraction protocols that were applied. This was necessary to support extraction of DNA from the low-biomass Atacama samples (we initially attempted to use the same extraction protocol but achieved insufficient DNA yield). The same amplification protocol, including PCR primers, was used for both studies, and both studies used Illumina sequencing instruments. While differences in observed sample composition are expected to arise due to differences in extraction method (56), these differences have been shown to be generally smaller than the differences arising from biological effects (57, 58).

Community richness and diversity were quantified using phylogenetic diversity (PD) (59) and Shannon and Simpson indices within QIIME 1.9.1. Differences in community composition were calculated using weighted and unweighted UniFrac metrics (60), with communities rarefied to 17,212 sequences per sample, and PCoA and nonmetric multidimensional scaling (NMDS) were performed with QIIME. Weighted and unweighted UniFrac distance matrices were strongly correlated (Mantel *r* = 0.78, *P* < 0.001), so ordination plots are presented only for unweighted Unifrac data. Statistical significance corresponding to differences in beta diversity across discrete sample groupings was calculated using ANOSIM, and correlations between weighted and unweighted UniFrac distance and continuous environmental variables were tested using Mantel’s r statistic. BEST analysis was used to find the highest Spearman’s correlation value for comparisons between community dissimilarities and groups of environmental variables (37) by selecting all possible subsets of environmental variables given by the user and scaling and calculating the Euclidean distances for each subset (37). Spearman’s correlation coefficients were calculated using R (Core Development Team 2015), and corresponding *P* values were adjusted to compensate for the false-discovery rate (FDR [*q*]).

Network analysis was performed on sample OTUs. Prior to analysis, rare OTUs with abundances of less than 0.01% of the total number of OTUs were removed, resulting in a final subset of 1,293 OTUs. Co-occurrence of OTUs was defined based on their Spearman correlations using the WGCNA package (61). The nodes in each network represent OTUs, and the edges connecting the nodes represent correlations between OTU pairs. All *P* values were adjusted for multiple testing using the Benjamini and Hochberg FDR controlling procedure (62), as implemented in the multtest R package. The direct-correlation dependencies were distinguished using the network deconvolution method (63). Edges were pruned to keep only high-correlation coefficients and significant FDR-adjusted *P* values for correlations. The cutoff for presenting FDR-adjusted *P* values was 0.01, and the cutoff of correlation coefficients was found to be 0.81 for the global network through the random matrix theory (RMT) method (58). Network statistical properties were calculated at the sample level with the igraph R package and aggregated at each climate level. Graphical representations for the subnetwork arid, margin, and hyperarid data had RMT thresholds of 0.82, 0.92, and 0.84, respectively.

Some network statistics are impacted by community richness, and our three climate classes (arid, margin, and hyperarid) differ in their levels of community richness. We therefore tested our conclusions for robustness with respect to differences in richness by reducing our higher-richness samples (from the arid and margin sites) to the richness level of the hyperarid sites and recomputing our network statistics. The effect of richness reduction was tested by rarefying the initial OTU table 500 times to 300 reads per sample and removing rare OTUs using the same method as that described before. Networks were created and statistics were averaged across all 500 repeats using R. These “reduced-richness” arid soil and margin soil samples had median alpha diversity values (130.5 and 119.3 species observed, respectively) similar to the original alpha diversity values determined for hyperarid samples (122 species observed). Median values for “reduced-richness” network properties for arid and margin samples were calculated using R (Table 2).

### Accession number(s).

All sequences have been archived in the Qiita database (http://qiita.microbio.me) under study identifier (ID) 10360 and in the European Nucleotide Archive of the European Bioinformatics Institute (EMBL-EBI) under accession number ERP019482.
