# Supplementary material for: Significant Impacts of Increasing Aridity on the Arid Soil Microbiome
Source: mSystems. 2017 May 30;2(3):e00195-16. doi: 10.1128/mSystems.00195-16 (PMC5451488; doi:10.1128/mSystems.00195-16)
Supplement: TABLE S1 [file sys003172106st1.pdf]

Table S1. Site GPS coordinates (WGS84 datum) environmental characterization.

Site ID	Latitude (°S)	Longitude (°W)	Elevation (masl)	pH	EC (dS m^{-1})	AvgT (°C±SD)	Avg SoilRH (%±SD)	HighT (°C±SD)	LowT (°C±SD)	High Soil RH (%±SD)	Low SoilRH (%±SD)	PercSoil RH100 (%)	Plant Cover (%)	Aridity Class
**Yungay Transect**														
YUN1242	24.14112	70.31242	1242	9.00	1.845	23.6±3.3	20.90±3.11	27.6±2.0	18.3±2.0	36.46±0.63	16.96±0.38	0	0	**Hyperarid**
YUN2029	24.59866	70.31483	2029	7.89	0.067	17.9±4.1	28.79±8.34	24.2±0.7	10.2±1.4	52.55±0.72	21.76±1.47	0	0	**Hyperarid**
YUN1005	24.0923	69.9665	1005	7.60	2.27	22.6±3.3	20.70±3.12	26.3±1.5	16.5±1.4	31.00±0.89	16.34±0.39	0	0	**Hyperarid**
YUN1609	24.14417	69.44242	1609	8.00	0.427	21.5±3.4	17.18±6.63	25.4±1.3	16.1±2.0	50.40±1.21	12.53±0.26	0	0	**Hyperarid**
YUN3153	24.23398	68.85686	3153	7.60	2.26	12.5±4.9	59.69±17.84	19.6±1.2	3.5±1.6	93.15±0.23	32.12±0.33	0	0	**Hyperarid**
YUN3008	24.22445	68.7516	3008	7.71	2.205	11.6±5.6	70.89±22.63	19.9±2.0	2.0±1.9	100.00±0.00	35.78±0.75	29.1	0	**NA**
YUN3184	24.22214	68.69441	3184	7.70	2.235	14.1±4.8	26.97±7.32	22.0±1.0	5.3±1.0	45.03±0.87	15.84±0.35	0	0	**NA**
YUN3346	24.18467	68.59204	3346	7.10	0.044	15.9±4.9	87.32±13.96	24.0±1.4	6.9±1.6	100.00±0.00	57.80±0.42	33.1	0.01	**Arid**
YUN3259	24.17038	68.53413	3259	8.10	0.133	15.3±5.1	93.57±10.17	23.7±1.4	6.3±1.5	100.00±0.00	69.04±0.77	64.7	2.4	**Arid**
YUN3428*	24.34139	68.35242	3428	7.20	0.023	13.6±5.8	99.99±0.08	21.1±1.8	4.1±1.0	100.00±0.00	99.91±0.21	99.4	8.8	**Arid**
YUN3533	24.37951	68.36079	3533	8.00	0.024	11.4±5.2	100.00±0.08	19.2±1.2	3.5±1.3	100.00±0.00	99.93±0.21	99.9	8.6	**Arid**
YUN3856	24.44621	68.29649	3856	7.43	0.029	9.5±5.9	99.44±2.99	17.5±2.2	0.5±1.3	100.00±0.00	76.39±1.30	93.7	3.1	**Arid**
**Baquedano Transect**														
BAQ895	23.40284	69.98714	895	7.88	2.945	19.2±3.6	32.22±3.07	24.7±1.2	12.6±0.9	41.02±0.94	27.00±0.64	0	0	**Hyperarid**
BAQ1370	23.35399	69.67095	1370	7.98	6.08	22.6±3.6	16.17±2.61	28.2±1.4	17.7±1.6	22.63±0.68	11.95±0.25	0	0	**Hyperarid**
BAQ1552	23.40907	69.43716	1552	7.65	1.839	22.6±3.8	15.75±3.88	27.5±1.5	16.0±2.0	33.41±0.97	11.88±0.45	0	0	**Hyperarid**
BAQ2838	22.65381	68.57623	2838	8.36	0.212	19.1±4.9	44.74±27.98	25.0±2.5	10.7±2.4	100.00±0.00	16.65±0.46	11.3	0	**Margin**
BAQ2420	22.91801	68.3532	2420	9.36	0.075	22.5±5.3	82.54±19.89	28.3±2.0	13.3±1.7	100.00±0.00	41.13±0.89	46.8	0	**Margin**
BAQ2462	22.97577	68.41652	2462	8.35	0.506	21.8±5.2	69.08±19.99	29.0±1.1	13.0±1.6	98.40±0.22	32.56±0.42	0.2	0	**Margin**
BAQ2687	22.91819	68.05283	2687	8.35	0.131	18.2±4.7	73.21±29.33	25.0±1.5	10.7±1.3	100.00±0.00	20.87±0.53	43.5	0.1	**Arid**
BAQ3473	22.91237	67.94507	3473	7.93	0.42	13.5±5.3	82.05±18.05	20.7±2.4	5.1±1.5	100.00±0.00	42.09±0.39	35.4	1.2	**Arid**
BAQ4166	22.92925	67.86743	4166	7.22	0.084	9.4±5.0	100.00±0.09	17.5±1.9	1.4±1.0	100.00±0.00	100.00±0.00	100.0	7.1	**Arid**
BAQ4697	22.95144	67.68925	4697	7.44	0.055	Site was destroyed and data logger lost; no data available							0.1	**Arid**

masl, meters above sea level; EC, Electrical Conductivity; average soil temperature (AvgT); average soil relative humidity (AvgSoilRH);

maximum soil temperature averaged over 7 days (HighT); minimum soil temperature averaged over 7 days (LowT); maximum soil relative

humidity averaged over 7 days (HighSoilRH); minimum soil relative humidity averaged over 7 days (Low SoilRH); Percent soil relative humidity 100 (PercSoilRH100: the percent of all values collected at each study site that were at 100%). Values based on data collected at 2 hour intervals from March 2012 through January 2015 from pit 1 at each site at a depth of 20 cm. *YUN3428 represents only values collected from March 2012 through December 2013 due to battery failure. Plant cover (%) measured in March 2012 at time of sampling; aridity class as defined in the text. NA; salt levels influenced by proximity to Salar de Imilac and thus could not be used for aridity classification.
