# Supplementary material for: Significant Impacts of Increasing Aridity on the Arid Soil Microbiome
Source: mSystems. 2017 May 30;2(3):e00195-16. doi: 10.1128/mSystems.00195-16 (PMC5451488; doi:10.1128/mSystems.00195-16)
Supplement: TABLE S2 [file sys003172106st2.pdf]

Table S2. Microbial community diversity indices, DNA extract concentrations, and soil organic carbon content for 40 samples included in the microbial analysis.

Transect	Soil Pit ID	Shannon	Simpson	DNA extract concentration (ng ul^{-1})	SOC (mg C g dry $soil^{-1}$)
Baquedano	BAQ2838.1	8.742	0.994	0.467	0.353
	BAQ2838.2	8.747	0.994	0.309	0.26
	BAQ2838.3	7.938	0.989	0.145	0.369
	BAQ2420.1.2	8.693	0.992	0.108	0.166
	BAQ2420.2	7.764	0.986	0.089	0.337
	BAQ2420.3	7.711	0.989	0.083	0.574
	BAQ2462.1	7.653	0.981	1.166	0.173
	BAQ2462.2	7.029	0.978	0.049	0.331
	BAQ2462.3	7.566	0.988	0.066	0.634
	BAQ2687.1	9.304	0.995	1.223	0.554
	BAQ2687.2	6.800	0.977	0.251	0.35
	BAQ2687.3	8.482	0.993	1.176	0.4
	BAQ3473.1	9.540	0.993	16.374	3.675
	BAQ3473.2	9.694	0.996	5.17	0.838
	BAQ3473.3	8.666	0.991	0.91	16.449*
	BAQ4166.1.2	9.302	0.995	16.086	2.085
	BAQ4166.2	9.453	0.996	2.958	3.692
	BAQ4166.3	9.420	0.995	28.92	2.271
Yungay	YUN1242.1	4.563	0.905	0.228	0.226
	YUN1242.3	5.491	0.948	0.05	0.452
	YUN2029.2	6.035	0.957	0.232	0.241
	YUN1005.3	4.828	0.918	0.028	0.223
	YUN1609.1	4.340	0.788	0.031	0.361
	YUN3153.2	6.671	0.970	0.493	0.185
	YUN3153.3	6.189	0.962	0.22	0.391
	YUN3346.1	7.023	0.970	0.314	0.429
	YUN3346.2	7.711	0.984	0.318	0.666
	YUN3346.3	7.573	0.967	1.218	0.387
	YUN3259.1.2	8.023	0.987	1.543	0.419
	YUN3259.2	9.629	0.997	1.935	0.342
	YUN3259.3	8.620	0.984	3.53	0.539
	YUN3428.1	9.096	0.993	1.764	0.623
	YUN3428.2	9.155	0.994	1.988	0.621
	YUN3428.3	9.311	0.995	2.782	0.658
	YUN3533.1.2	9.639	0.996	3.21	0.521
	YUN3533.2	6.921	0.893	9.236	0.773
	YUN3533.3	9.288	0.994	3.642	0.664
	YUN3856.1.2	7.823	0.959	6.23	0.713
	YUN3856.2	8.993	0.994	4.178	0.404
	YUN3856.3	9.073	0.995	6.695	0.988

SOC: Soil organic carbon

* Nomadic grazing in this region contributed elevated allochthonous SOC levels at this site from animal feces.
