# Supplementary figures and images for: Significant Impacts of Increasing Aridity on the Arid Soil Microbiome
Source: mSystems. 2017 May 30;2(3):e00195-16. doi: 10.1128/mSystems.00195-16 (PMC5451488; doi:10.1128/mSystems.00195-16)

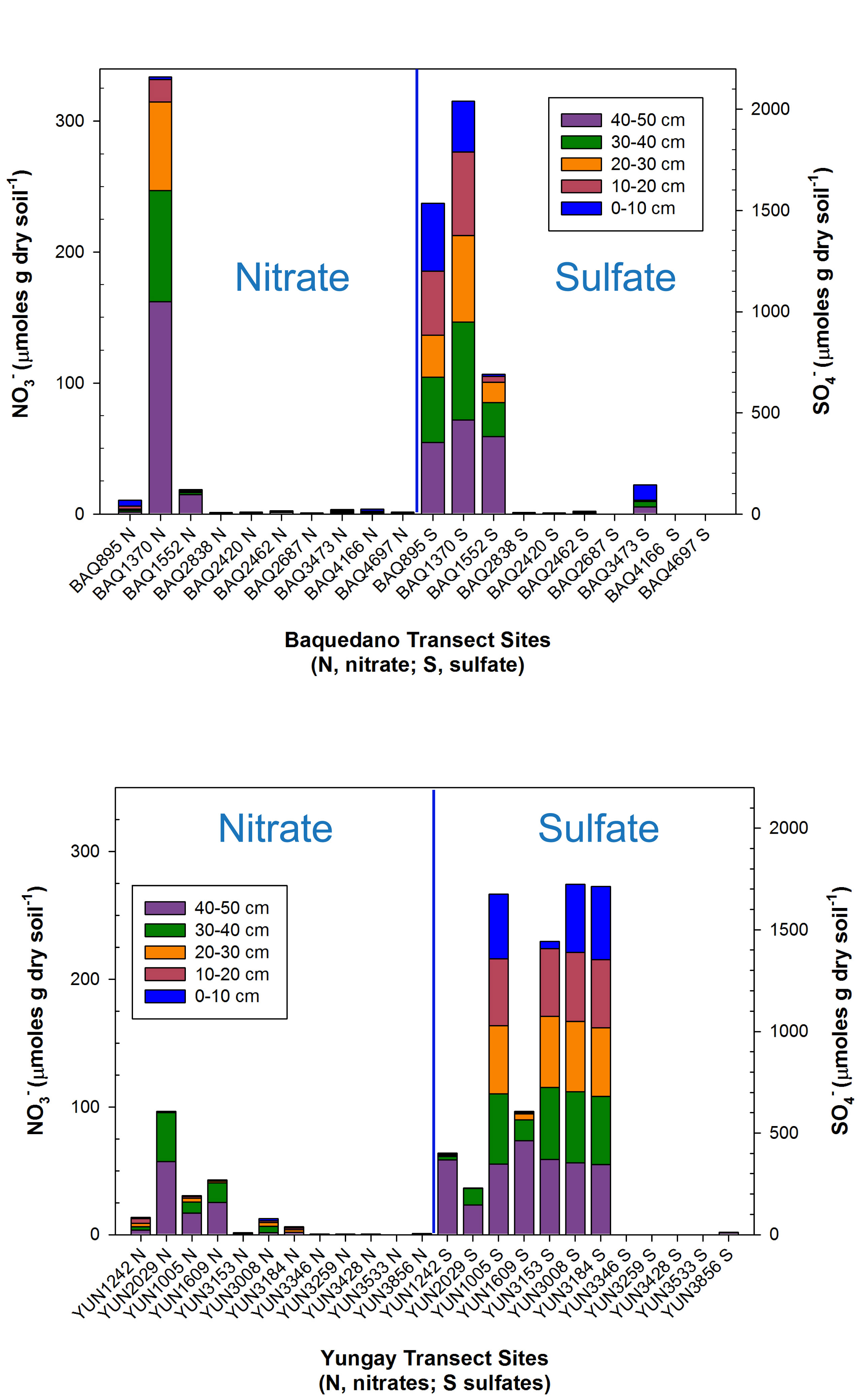

Supplement: FIG S1 [file sys003172106sf6.jpg]
