# Supplementary material for: Significant Impacts of Increasing Aridity on the Arid Soil Microbiome
Source: mSystems. 2017 May 30;2(3):e00195-16. doi: 10.1128/mSystems.00195-16 (PMC5451488; doi:10.1128/mSystems.00195-16)
Supplement: TABLE S3 [file sys003172106st3.pdf]

Table S3. UniFrac Mantel's correlation values between UniFrac distance matrix and the pairwise dissimilarity matrix for each environmental variable.

Environmental Variable	Mantel's r	p - value	*q* - value
**Unweighted Unifrac Analysis**			
High Soil RH	0.663	0.0001	0.0001
Elevation*	0.651	0.0001	0.0001
Average Soil RH	0.625	0.0001	0.0001
Low Soil Temperature	0.541	0.0001	0.0001
Electrical Conductivity	0.496	0.0001	0.0001
Percent Days at 100% Soil RH	0.479	0.0001	0.0001
Average Soil Temperature	0.477	0.0001	0.0001
High Soil Temperature	0.377	0.0001	0.0001
Low Soil RH	0.320	0.0002	0.0003
Plant Cover (%)	0.184	0.016	0.018
pH	0.176	0.009	0.010
Soil Organic Carbon	0.151	0.052	0.055
**Weighted Unifrac Analysis**			
Elevation*	0.596	0.0001	0.0001
Average Soil RH	0.574	0.0001	0.0001
High Soil RH	0.566	0.0001	0.0001
Electrical Conductivity	0.458	0.0001	0.0001
Low Soil Temperature	0.458	0.0001	0.0001
Percent Days at 100% Soil RH	0.454	0.0001	0.0001
Average Soil Temperature	0.394	0.0001	0.0001
Low Soil RH Minimum	0.362	0.0001	0.0001
Soil Organic Carbon	0.309	0.0009	0.001
High Soil Temperature	0.304	0.0001	0.0001
Plant Cover (%)	0.233	0.004	0.004
pH	0.108	0.063	0.063

q-value: false discovery rate (FDR) corrected p - values; RH, relative humidity

*Elevation is conflated with collection site.
