# Supplementary material for: Significant Impacts of Increasing Aridity on the Arid Soil Microbiome
Source: mSystems. 2017 May 30;2(3):e00195-16. doi: 10.1128/mSystems.00195-16 (PMC5451488; doi:10.1128/mSystems.00195-16)
Supplement: TABLE S4 [file sys003172106st4.pdf]

Table S4. Spearman's rank correlations between average soil relative humidity and relative abundance of taxa at the domain and phylum level.

**Phylogenetic Classification**	**Taxon**	**Spearman's rank correlation coefficient (r_s)**	**P - value**	***q* - value**
**Domain**				
	Archaea	0.757	0.002	0.005
**Phylum**				
	Acidobacteria	0.925	< 2.2e-16	2.31E-15
	Proteobacteria	0.857	0.000009	0.00005
	Planctomycetes	0.857	0.000009	0.00005
	Verrucomicrobia	0.842	0.00008	0.0003
	Euryarchaeota	0.818	0.0002	0.0007
	Crenarchaeota	0.757	0.002	0.005
	Nitrospirae	0.764	0.001	0.003
	Elusimicrobia	0.702	0.004	0.008
	Fibrobacteres	0.665	0.007	0.013
	FBP	0.655	0.008	0.014
	Gemmatimonadetes	0.65	0.011	0.018
	Armatimonadetes	0.577	0.024	0.036
	AD3	0.559	0.030	0.042
	TM7	0.528	0.043	0.056
	Firmicutes	0.332	0.226	0.264
	Bacteroidetes	0.3	0.277	0.306
	Cyanobacteria	0.232	0.404	0.424
	GAL15	-0.061	0.828	0.828
	Chloroflexi	-0.464	0.083	0.103
	Actinobacteria	-0.95	< 2.2e-16	2.31E-15

q-value: false discovery rate (FDR) corrected p - values
