# Supplementary material for: Significant Impacts of Increasing Aridity on the Arid Soil Microbiome
Source: mSystems. 2017 May 30;2(3):e00195-16. doi: 10.1128/mSystems.00195-16 (PMC5451488; doi:10.1128/mSystems.00195-16)
Supplement: TABLE S5 [file sys003172106st5.pdf]

Table S5. Distribution of soil genera commonly associated with nitrogen cycling activities and correlation with average soil relative humidity.

Genus	Phylum/ Subphylum	Range in relative abundance (%)	Spearman's rank correlation coefficient (r_s)[a]	*P* - value	q - value	Functional Association
Nitrospira	Nitrospirae	0 – 0.1	0.805	0.0003	0.002	NO_2 oxidation
Bradyrhizobium	α-Proteobacteria	0 – 1.2%	0.787	0.0005	0.002	N_2 fixation
Nitrososphaera	Crenarchaeota	0 - 2.6	0.757	0.002	0.006	NH_3 oxidation
Mesorhizobium	α-Proteobacteria	0 – 0.15	0.707	0.003	0.007	N_2 fixation
Frankia	Actinobacteria	0 – 0.1	0.376	0.167	0.301	N_2 fixation
Rhizobium	α-Proteobacteria	0 – 0.09	0.311	0.259	0.389	N_2 fixation
Azospirillum	α-Proteobacteria	0 – 0.007	0.266	0.339	0.432	N_2 fixation
Nitrosovibrio	β-Proteobacteria	0 – 0.04	0.243	0.384	0.432	NH_3 oxidation
Sinorhizobium	α-Proteobacteria	0 – 0.04	0.128	0.649	0.649	N_2 fixation
Nitrobacter	α-Proteobacteria	ND				NO_2 oxidation
Nitrosomonas	β-Proteobacteria	ND				NH_3 oxidation
Nitrosospira	β-Proteobacteria	ND				NH_3 oxidation

a: Spearman's rank correlation of soil genera with average soil relative humidity.

q-value: false discovery rate (FDR) corrected p – values; ND, Taxa not detected.
